# *NtAGL6* of *Narcissus tazetta* var. *chinensis* Promotes Flowering via an Indirect Activation Pathway of *FT*

**DOI:** 10.3390/plants15162512

**Published:** 2026-08-20

**Authors:** Jinghua Wu, Lu Zhang, Lin Li, Zhizhong Zhang, Qiuyu Pan

**Affiliations:** College of Horticulture, Fujian Agriculture and Forestry University, No. 15 Shangxiadian Road, Cangshan District, Fuzhou 350002, China; zhanglummmm@163.com (L.Z.); lilnuli@163.com (L.L.); zhizhongzhang@fafu.edu.cn (Z.Z.); panqiuyuuuuu@163.com (Q.P.)

**Keywords:** floral organ development, flowering regulation, indirect regulation, *Narcissus tazetta* var. *chinensis*, *NtAGL6*

## Abstract

*Narcissus tazetta* var. *chinensis* is a monocot with unique floral morphology and heat-induced flowering. In *Arabidopsis*, the MADS-box gene *AGL6* promotes flowering by positively regulating *FT* expression, but whether this regulatory mechanism is conserved in monocots remains unknown. Here, we cloned the *AGL6* homolog *NtAGL6* from *N. tazetta* var. *chinensis*, analyzed its sequence, phylogeny, expression pattern, and promoter cis-elements, and performed ectopic expression in wild-type and *ap1* mutant *Arabidopsis*. Yeast one-hybrid and transient expression assays were used to test its interaction with the *NtFT1* promoter. *NtAGL6* was specifically expressed in floral organs and during flower bud differentiation, and its promoter contained heat shock and gibberellin response elements. Ectopic expression of *NtAGL6* promoted flowering in *Arabidopsis* and upregulated *FT*, *SOC1*, *LFY*, and *AP1*, and partially rescued petal and sepal defects in the *ap1* mutant. Although *NtAGL6* activated *NtFT1* promoter activity in transient assays, yeast one-hybrid assays detected no direct binding. Together, these results indicate that *NtAGL6* promotes flowering and affects A-class floral organ identity, but likely regulates *FT* through an indirect pathway in this monocot. This study provides new insights into the functional divergence of *AGL6* in flowering regulation in monocots.

## 1. Introduction

Flowering is a critical developmental transition from vegetative to reproductive growth in plants. This process is governed by a complex genetic regulatory network that integrates multiple signals, including photoperiod, temperature, hormones, and plant age, to initiate floral transition at the appropriate time [1,2]. Epigenetic mechanisms, including histone modifications and chromatin remodeling, play critical roles in integrating environmental signals to regulate flowering time [3]. In *Arabidopsis*, *FT* and *SOC1* serve as central integrators of these signaling pathways. They activate the floral meristem identity genes *LFY* and *AP1*, which subsequently initiate floral development [4].

Floral organ morphogenesis represents another key aspect of the flowering process. Based on classical genetic studies in *Arabidopsis* and snapdragon (*Antirrhinum majus*), the ABCDE model of floral organ development has been largely established [5,6,7,8]. Through continuous refinement, this model has become a general framework for understanding floral development [9]. According to this model, the identities of sepals, petals, stamens, carpels, and ovules are determined by the combinatorial actions of A + E, A + B + E, B + C + E, C + E, and D + E class genes, respectively. Except for *AP2*, the B, C, D, E, and A-class gene *AP1* all belong to the MADS-box gene family [7,10]. MADS-box genes encode a family of transcription factors that contain a highly conserved MADS domain, which mediates sequence-specific DNA binding and is essential for protein–protein interactions [11]. These genes exhibit diverse and distinct functions in floral organ morphogenesis, meristem determinacy, flowering time regulation, and fruit and seed development [12,13].

The MADS-box gene family contains an ancient subfamily, *AGL6*, whose origin can be traced back to the common ancestor of angiosperms and gymnosperms [14,15]. In the dicot model plant *Arabidopsis*, *AGL6* and its paralogs are primarily involved in flowering time regulation and gametophyte development. In *Arabidopsis*, *AGL6* promotes flowering by positively regulating *FT* expression and repressing the *FLC*/*MAF* clade genes [16,17,18]. This finding positions *AGL6* as an important node linking temperature signals to the flowering decision center. Moreover, *AGL6* has been shown to regulate *FT* indirectly by repressing *ELF3*, indicating that its control of flowering involves multiple regulatory layers [19].

In monocots, however, the functional landscape of *AGL6* homologs appears more complex and diverse. In rice, the ABC model of floral organ development has been well established and provides a framework for understanding floral development in other monocots [20]. In rice (*Oryza sativa*), *OsMADS6* and *OsMADS17* are involved not only in floral organ identity determination but also in inflorescence meristem development and grain morphology [21,22]. In maize (*Zea mays*), the bearded-ear gene plays a critical role in inflorescence development [23]. In wheat (*Triticum aestivum*), *AGL6* homologs are indispensable for normal stamen development and can serve as operable targets for spike meristem development [24,25]. In orchids, *AGL6* genes have undergone significant diversification, with different copies regulating the formation of sepals, petals, lips, and columns [26,27]. Notably, most of these studies in monocots have focused on floral organ morphogenesis. In contrast, the role of *AGL6* in flowering time regulation in monocots, as well as its regulatory mode toward *FT*, has rarely been reported. Whether the regulatory mode of *AGL6* on *FT* expression observed in *Arabidopsis* is conserved in monocots remains to be directly tested.

*Narcissus tazetta* var. *chinensis* Roem is a perennial bulbous herb in the family Amaryllidaceae and provides a unique material for addressing the above questions. First, its floral organs exhibit distinctive structures. In the single-petal cultivar ‘Jinzhan Yintai’, a characteristic corona is present, which is a floral organ unique to the genus *Narcissus*. The molecular mechanism underlying its development remains unclear. The double-petal cultivar ‘Yulinglong’ shows complete petaloidy of stamens, offering valuable material for studying the molecular basis of double-flower formation. Second, *N. tazetta* var. *chinensis* is characterized by high temperature-induced floral bud differentiation, which requires a temperature above 25 °C [28]. Further studies have shown that under high temperature conditions, the expression of *FT* homologs in this species is upregulated in parallel with the progress of floral bud differentiation [29]. This feature contrasts sharply with the vernalization pathway in the model plant *Arabidopsis*, suggesting that *N. tazetta* var. *chinensis* may have evolved a unique mechanism for temperature perception and flowering regulation. In recent years, transcriptomic analyses of *Narcissus* floral organs have preliminarily revealed the regulatory networks of color and scent metabolic pathways [30]. Cloning and heterologous functional validation of *FT* homologs have also been reported [29]. However, systematic functional characterization of key regulatory genes such as *AGL6* has not yet been performed.

It should be noted that a genetic transformation system for *N. tazetta* var. *chinensis* is not yet available. As a bulbous flower with a growth cycle of several years, its stable transformation efficiency is extremely low, and transient transformation systems are also suboptimal. This severely limits the direct verification of gene functions in *Narcissus*. Therefore, this study adopted a strategy of using model plants as functional validation platforms. Using *N. tazetta* var. *chinensis* as the gene donor, we performed systematic preliminary functional analysis of *NtAGL6* through heterologous expression in *Arabidopsis* and transient expression in *Nicotiana benthamiana*. Although heterologous systems cannot fully recapitulate all details of the endogenous regulatory network in *Narcissus*, this strategy has been widely applied and recognized in horticultural plants that are difficult to transform genetically, such as lily, tulip, orchid, and mei flower (*Prunus mume*) [31,32,33]. Under current technical constraints, this approach can provide valuable evidence and clues for gene function.

Based on the above background, this study focused on two core questions: (1) Does *NtAGL6* possess the capacity to promote flowering, and can it partially compensate for the loss of A-class floral organ identity function when ectopically expressed in a heterologous system? (2) Does *NtAGL6* regulate *NtFT1* through a regulatory mode similar to that of *Arabidopsis AGL6* on *FT*, or does it employ a distinct mechanism? To address these questions, we cloned the *AGL6* homolog *NtAGL6* from *N. tazetta* var. *chinensis*. Through systematic expression analysis, promoter characterization, heterologous functional validation in *Arabidopsis*, and investigation of its regulatory mechanism on the *NtFT1* promoter, we preliminarily dissected its potential roles in floral organ development and flowering induction. The results of this study are expected to provide molecular evidence for understanding the functional evolution of *AGL6* genes in the regulation of floral development in monocots, and to lay a foundation for the eventual elucidation of the functional network of *AGL6* in *Narcissus*.

## 2. Results

### 2.1. Cloning and Sequence Characterization of NtAGL6

**Figure 1:**
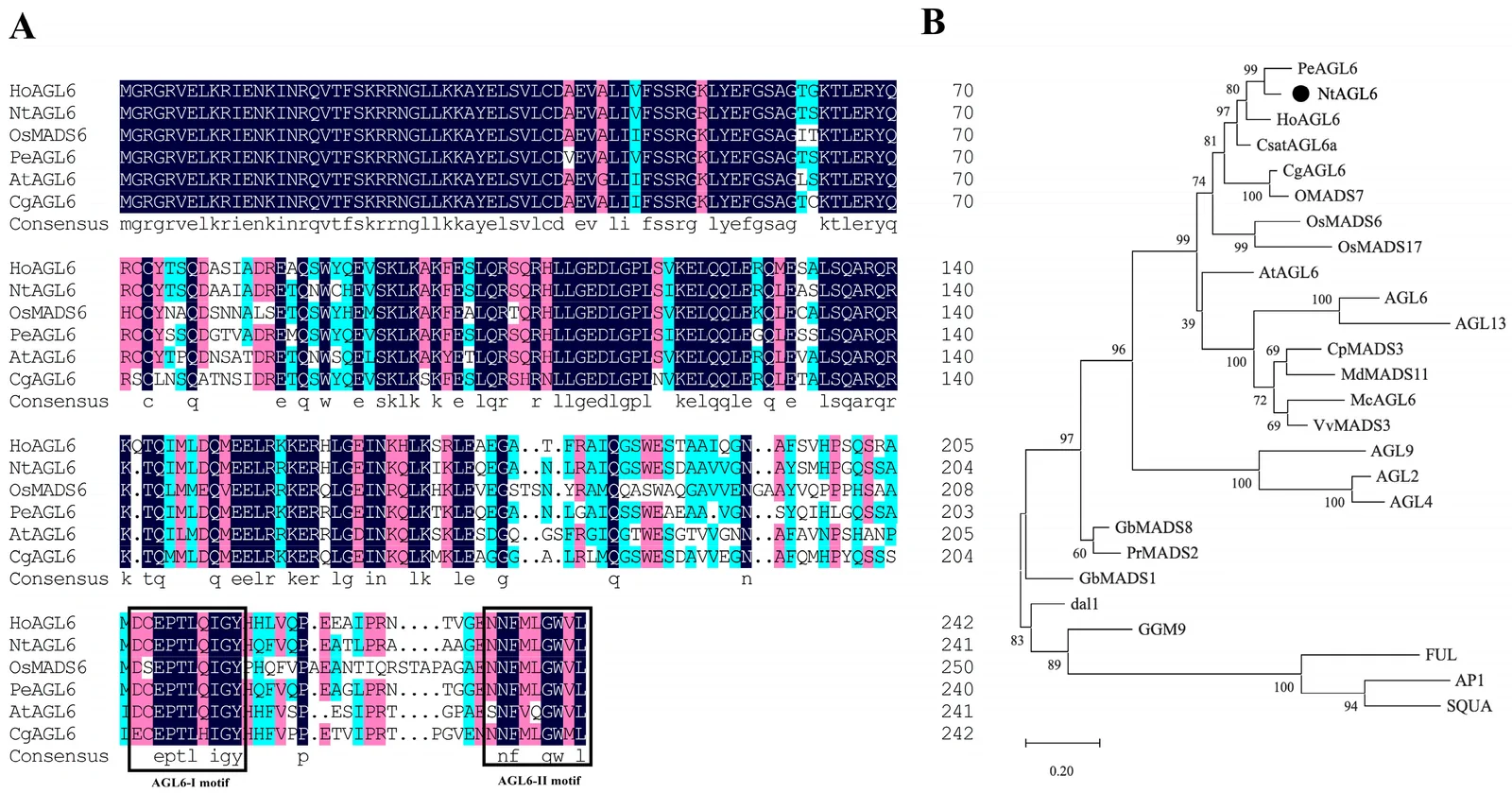
Multiple sequence alignment (**A**) and phylogenetic analysis (**B**) of NtAGL6 with other MADS-box proteins. The phylogenetic tree was constructed using the neighbor-joining method with 1000 bootstrap replicates.

A full-length ORF of a MADS-box gene was cloned from the petal cDNA of *N. tazetta* var. *chinensis* ‘Jinzhan Yintai’. The ORF was 726 bp in length, encoding 241 amino acids, and was named *NtAGL6*. Multiple sequence alignment showed that the NtAGL6 protein contained a highly conserved MADS domain (approximately 60 amino acids) at the N-terminus, as well as the AGL6-I motif and the AGL6-II motif at the C-terminus, together with a typical C-terminal sequence of AGL2-like proteins (Figure 1A). These characteristic motifs clearly assigned *NtAGL6* to the *AGL6-I-ZAG* subclade. Phylogenetic analysis showed that NtAGL6 clustered with AGL6 proteins from other monocots (such as orchid, hyacinth, and rice) in the same clade, and was most closely related to AGL6 from species of the Amaryllidaceae family. In contrast, NtAGL6 was clearly separated from members of other MADS-box subfamilies, such as AP1 and SEP (Figure 1B).

### 2.2. Expression Patterns of NtAGL6 in Different Tissues and Flower Bud Differentiation Stages of N. tazetta var. chinensis

**Figure 2:**
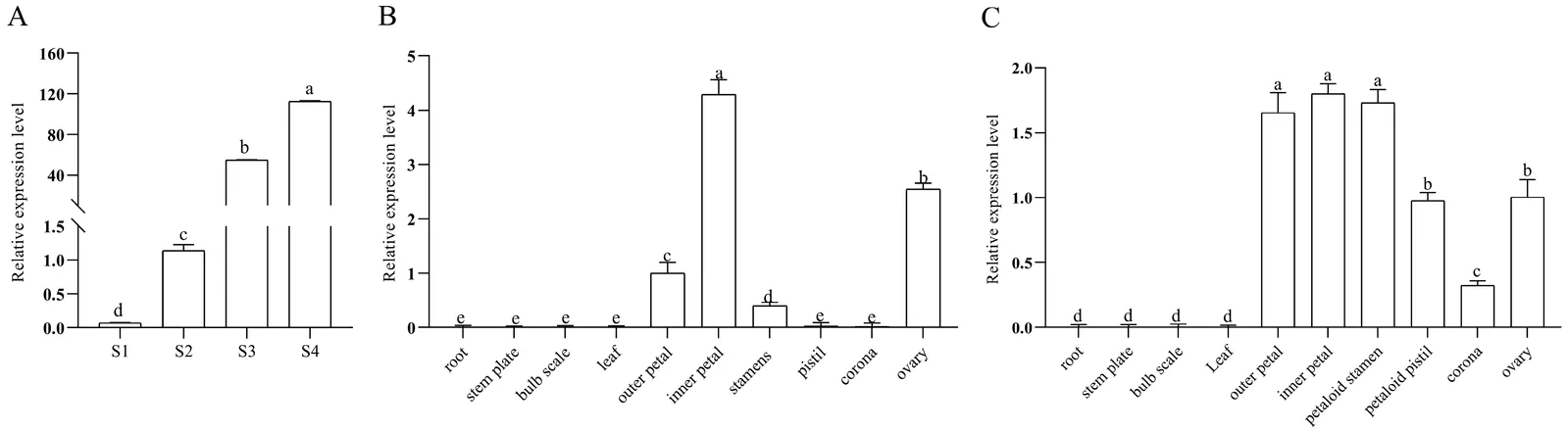
Expression profiles of *NtAGL6* in *N. tazetta* var. *chinensis*. (**A**) Expression of *NtAGL6* during flower bud differentiation. S1: leaf bud stage; S2: inflorescence primordium formation stage; S3: floral primordium formation stage; S4: floral organ formation stage. (**B**) Expression of *NtAGL6* in different tissues of single-flower cultivar. (**C**) Expression of *NtAGL6* in different tissues of double-flower cultivar. The different lowercase letters indicate significant differences at 0.05 level (*p* < 0.05).

At the leaf bud stage (S1), the expression level of *NtAGL6* was relatively low. After the transition to the inflorescence primordium formation stage (S2), the expression level increased significantly, reaching approximately 6.8-fold that at S1 (*p* < 0.01). The expression remained at a relatively high level during the floral primordium formation stage (S3) and the corolla formation stage (S4) (Figure 2A). This expression trend suggested that *NtAGL6* may play a role in floral transition and floral organ morphogenesis.

Tissue-specific expression analysis showed that in the single-petal cultivar ‘Jinzhan Yintai’, no expression signal of *NtAGL6* was detected in vegetative organs, including root, basal plate, scale, and leaf. Expression was detected exclusively in floral organs (Figure 2B). Among the floral organs, the expression level was highest in the inner petals, followed by the ovary (59.44% of that in the inner petals). Expression in stamens was relatively low (9.23% of that in the inner petals), while only very low levels were detected in pistils and corona (5.11% and 3.34% of that in the inner petals, respectively).

In the double-petal cultivar ‘Yulinglong’, *NtAGL6* was also not expressed in vegetative organs (Figure 2C). However, its expression level in petalized stamens was 4.37-fold higher than that in stamens of the single-petal cultivar, and its expression in petalized pistils was 40.28-fold higher than that in pistils of the single-petal cultivar.

### 2.3. Subcellular Localization of NtAGL6 Protein

**Figure 3:**
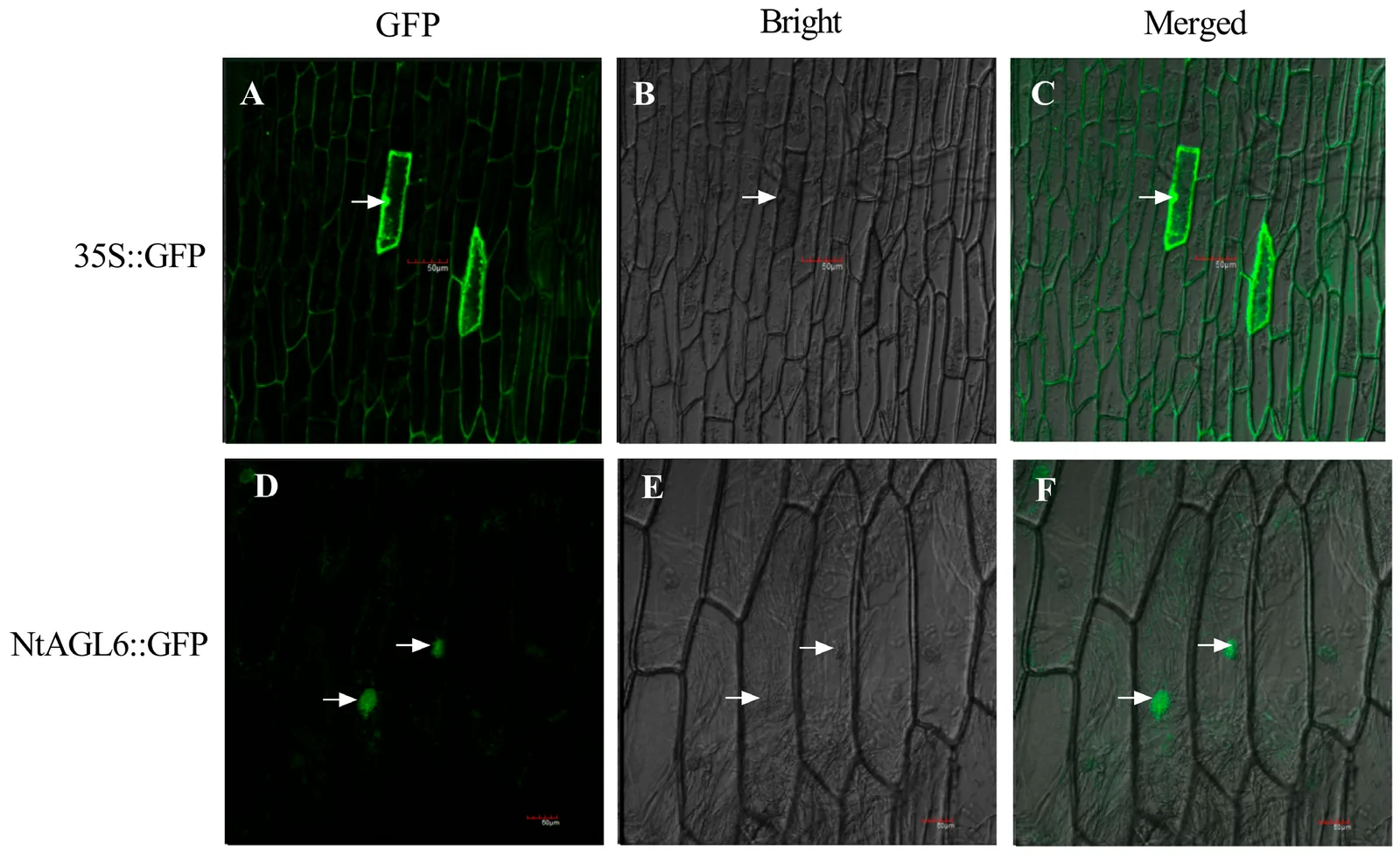
Subcellular localization of NtAGL6 protein. (**A**–**C**) GFP control: green fluorescence distributed throughout the entire cell. (**D**–**F**) NtAGL6-GFP fusion protein: green fluorescence concentrated in the nuclear region. Arrows indicate the nuclear region. The experiment was performed using transient expression in onion (*Allium cepa*) epidermal cells.

Confocal laser scanning microscopy observation showed that the green fluorescence of the GFP control was evenly distributed throughout the entire cell, whereas the green fluorescence signal of the NtAGL6-GFP fusion protein was concentrated exclusively in the nuclear region (Figure 3). This result confirmed that the NtAGL6 protein is localized in the nucleus, consistent with its molecular characteristics as a transcription factor.

### 2.4. Activity of the NtAGL6 Promoter and Its Response to Environmental Signals

An 803 bp promoter sequence upstream of the *NtAGL6* start codon was obtained by genome walking. Cis-acting element prediction analysis showed that this sequence contained nine TATA-box core elements and seventeen CAAT-boxes, indicating that it possesses the basic structural features of a typical eukaryotic promoter. In addition, several cis-elements related to plant growth, development, and environmental responses were predicted, including the meristem regulatory element CAT-box, gibberellin-responsive elements GARE-motif (2 copies), methyl jasmonate-responsive elements CGTCA-motif (4 copies) and TGACG-motif (3 copies), abscisic acid-responsive elements ABRE (3 copies), auxin-responsive element AuxRR-core (1 copy), salicylic acid-responsive elements TCA-element (2 copies), and multiple MYB transcription factor binding sites. Notably, no typical low-temperature-responsive elements (LTRE) were detected in this promoter sequence, but multiple HSE elements (heat shock-responsive elements, 3 copies) were present.

**Figure 4:**
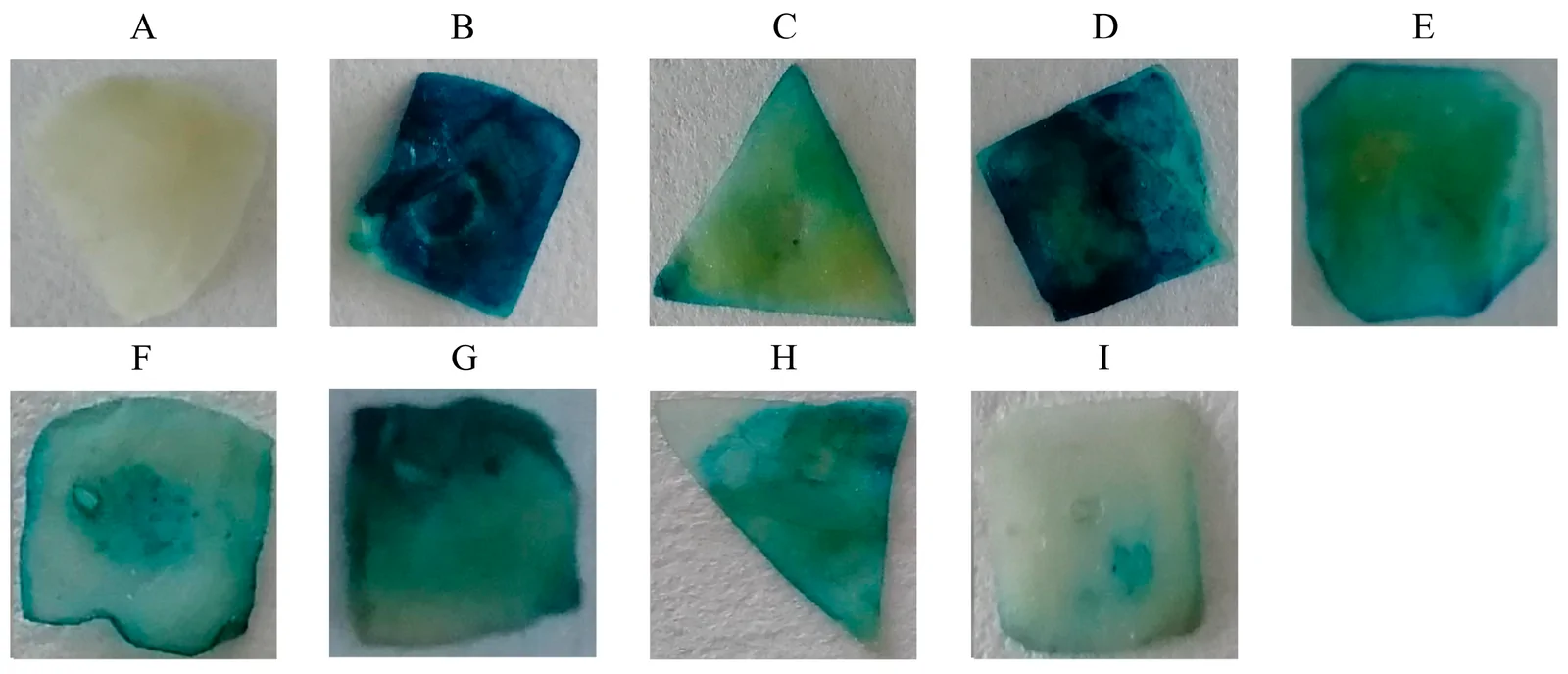
Histochemical GUS staining analysis of the *NtAGL6* promoter. (**A**) GV3101 (negative control). (**B**) pBI121 (positive control). (**C**) pBI121-NtAGL6::GUS. (**D**) High temperature treatment (35 °C). (**E**) Low temperature treatment (4 °C). (**F**) Spraying with 100 μmol·L^−1^ ABA. (**G**) Spraying with 100 μmol·L^−1^ GA_3_. (**H**) Spraying with 100 μmol·L^−1^ MeJA. (**I**) Spraying with water (control). GUS histochemical staining was performed on infiltrated leaves of *Nicotiana benthamiana*.

GUS histochemical staining showed that tobacco leaves infiltrated with pBI121-NtAGL6::GUS displayed distinct blue spots (Figure 4C), whereas negative control leaves infiltrated with *Agrobacterium* without the plasmid showed no blue signal (Figure 4A), confirming that the cloned upstream sequence possesses promoter activity.

Distinct differences in GUS staining intensity were observed under different treatment conditions (Figure 4D–H). GUS staining was markedly deepened in the high-temperature treatment group (35 °C, 24 h), with an increased number and intensity of blue spots. In contrast, the low-temperature treatment group (4 °C, 24 h) showed no obvious difference in staining intensity compared with the normal temperature control group (25 °C, normal culture). Moderate enhancement of GUS staining was observed following GA_3_ treatment, while the most pronounced induction was detected under high-temperature (35 °C) treatment. Moderate enhancement of GUS staining was also observed in the ABA and MeJA treatment groups, but to a lesser extent than in the GA_3_ treatment group. The positive control group (Figure 4B) exhibited the strongest blue staining, with a large coverage area and the deepest color.

### 2.5. Ectopic Expression of NtAGL6 in Wild-Type Arabidopsis Promotes Flowering

Three independent homozygous 35S::NtAGL6 transgenic lines (OE-1, OE-2, and OE-3) were used for flowering time measurement. All transgenic lines flowered earlier than the control, with the average days from transplanting to flowering reduced by 4.97 d compared with the empty vector control. The vegetative growth phase of transgenic plants was shortened, but no obvious morphological changes were observed in floral organs (including sepal, petal, stamen, and pistil number and morphology) after bolting, compared with the wild type.

**Figure 5:**
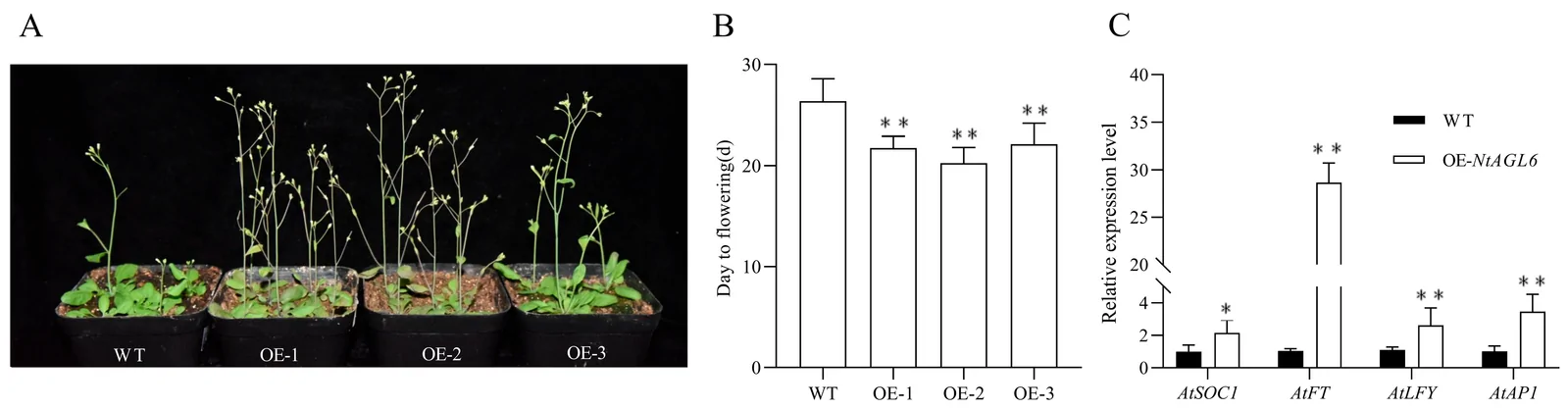
Functional analysis of *NtAGL6* by heterologous expression in *Arabidopsis*. (**A**) Phenotypic characterization of 35S::NtAGL6 overexpression *Arabidopsis* lines. (**B**) Days from transplanting to flowering. (**C**) Expression levels of flowering-related genes in transgenic plants. Asterisks indicate significant differences compared with the empty vector control (* *p* < 0.05, ** *p* < 0.01; Student’s *t*-test).

Compared with the empty vector control of the same age, *FT* expression showed the greatest upregulation (28.56-fold that of the control). *SOC1* was upregulated 2.15-fold, *LFY* 2.67-fold, and *AP1* 3.45-fold (Figure 5). The upregulation of *FT* was significantly greater than that of the other three genes.

### 2.6. Ectopic Expression of NtAGL6 Partially Rescues Floral Organ Defects in the ap1 Mutant

**Figure 6:**
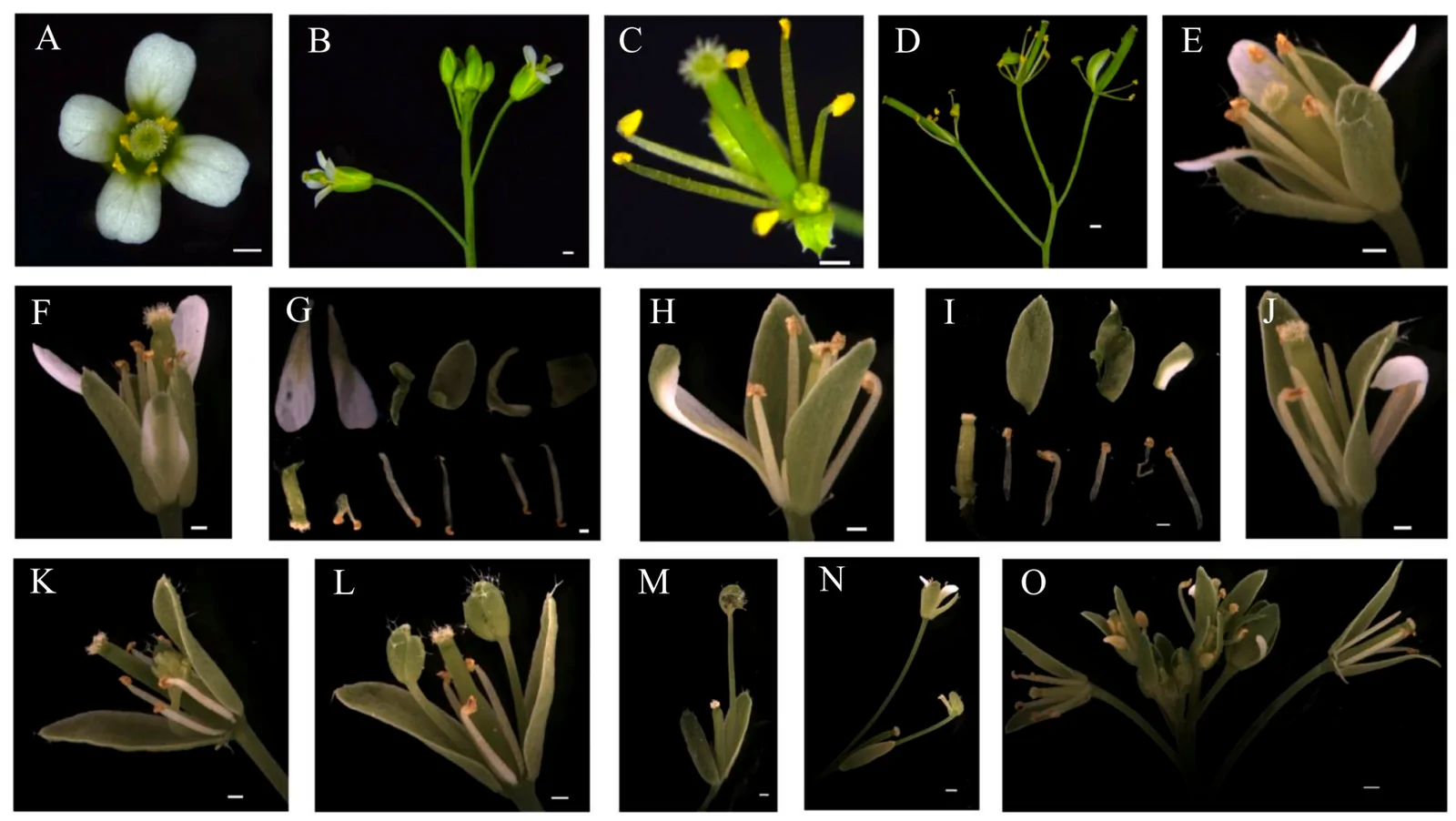
Phenotypes of *NtAGL6*-transgenic *ap1* mutant *Arabidopsis* plants. (**A**,**B**) Flowers and inflorescences of wild-type *Arabidopsis*. (**C**,**D**) Flowers and inflorescences of the ap1 mutant. (**E**–**J**) Transgenic plants rescued for petal and sepal defects of the ap1 mutant to various extents. (**H**,**I**) Sepal petalody and reduced stamen number. (**K**–**N**) Flowers bearing secondary flowers (flowers-within-flowers). (**O**) Terminal flower. Scale bars = 500 μm.

To test whether *NtAGL6* possesses a floral organ identity determination function, it was introduced into the *ap1* mutant, which exhibits severe defects in petal and sepal development. The *ap1* mutant control plants showed that sepals were partially transformed into bract-like structures and petals were completely absent (Figure 6C,D).

Five independent homozygous 35S::NtAGL6/ap1 transgenic lines were obtained, and phenotypic observation revealed that these lines exhibited varying degrees of phenotypic rescue (Figure 6E–J). Among them, three lines showed restored petals in some flowers, with 2–4 morphologically normal white petals produced (Figure 6E–G). Two lines showed partial rescue of sepal morphology, with bract-like structures transformed toward typical sepal morphology (Figure 6E,F). Sepal petalody was observed in two lines, where the first whorl organs displayed petal-like morphology (Figure 6H,I). Meanwhile, in flowers with partially restored petals and sepals, stamen number was reduced (normally 6, but only 4–5 stamens were observed in some flowers) (Figure 6I). Flowers-within-flowers (secondary flowers borne within a flower) were observed in three lines (Figure 6K–N), and a few terminal flowers borne at the apex of inflorescences were also observed (Figure 6O).

### 2.7. NtAGL6 Activates NtFT1 Promoter Activity but Does Not Bind Directly

**Figure 7:**
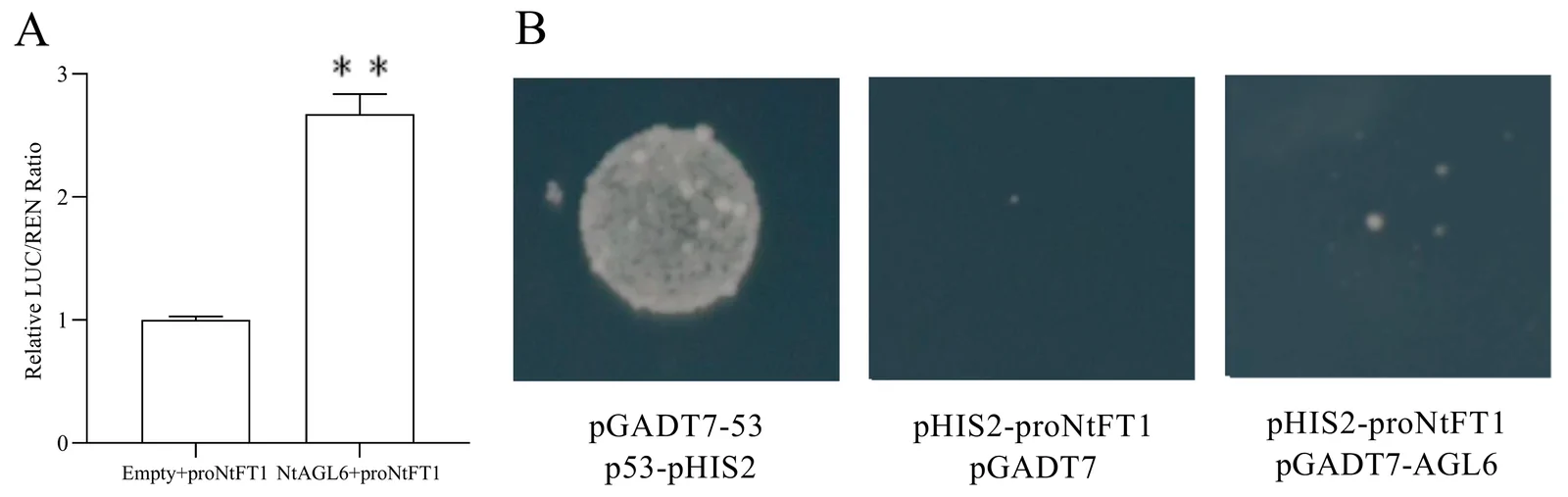
Regulatory analysis of *NtFT1* by NtAGL6. (**A**) Promoter activity measured by dual-luciferase transient expression assays. Statistical significance was determined using Student’s *t*-test (** *p* < 0.01). (**B**) Interaction of NtAGL6 with the *NtFT1* promoter in yeast cells. Yeast cells co-transformed with pHIS2-proNtFT1 and pGADT7-AGL6 showed no growth on selective medium, indicating no direct binding.

The transcriptional activation ability of *NtAGL6* on the *NtFT1* promoter was examined by dual-luciferase transient expression assays. In tobacco leaves co-infiltrated with the effector vector 35S::NtAGL6 and the reporter vector proNtFT1-LUC, the LUC/REN ratio was significantly higher than that of the control group co-infiltrated with the empty effector vector and the reporter vector, with a 2.57-fold increase (Figure 7A).

However, the yeast one-hybrid assay showed that yeast cells co-transformed with the bait vector pHIS2-proNtFT1 and the prey vector pGADT7-AGL6 failed to grow on SD/-Leu-Trp-His selective medium containing 30 mmol·L^−1^ 3-AT (Figure 7B), which was consistent with the growth status of the negative control (co-transformed with pGADT7 + pHIS2-proNtFT1). In contrast, the positive control (pGADT7-53 + p53-pHIS2) grew normally. These results indicated that NtAGL6 could not directly bind to the *NtFT1* promoter sequence.

## 3. Discussion

### 3.1. Molecular Identification of NtAGL6 and Its Position in the AGL6 Evolutionary Lineage

In this study, *NtAGL6* was cloned and identified from *N. tazetta* var. *chinensis*. The encoded protein contained an intact MADS domain, the *AGL6-I* and *AGL6-II* characteristic motifs, and an AGL2-like C-terminal sequence [34]. Phylogenetic analysis assigned it to the AGL6-I-ZAG subclade [35], where it clustered with AGL6 proteins from other monocots. Subcellular localization confirmed that it is a nuclear-localized transcription factor. These features confirmed that *NtAGL6* is a typical *AGL6* subfamily member in *N. tazetta* var. *chinensis*.

*AGL6* is one of the ancient subfamilies within the MADS-box family, with its origin predating the divergence of angiosperms and gymnosperms [14,15]. Two major clades, AGL6-I and AGL6-II, are commonly present in angiosperms, with members of the AGL6-I clade tending to be expressed in floral organs and involved in reproductive development [35]. The strictly floral organ-specific expression pattern of *NtAGL6* is consistent with the expression patterns of *AGL6* homologs previously reported in other monocots [23,24,26,36].

Thus, the sequence and expression characteristics of *NtAGL6* are clearly consistent with those of AGL6-I clade members in monocots. However, whether its function follows a known pattern or exhibits lineage-specific divergence requires further analysis from three aspects: expression characteristics, heterologous activity, and regulatory mode.

### 3.2. Expression Characteristics of NtAGL6 and Its Potential Association with High Temperature-Induced Flowering

The expression of *NtAGL6* was strictly organ-specific, with no signal detected in vegetative organs, including root, basal plate, scale, and leaf, but with signals detected exclusively in floral organs. This pattern is highly conserved in monocots [23,24,26,36], further supporting the view that AGL6-I clade members have become highly specialized as floral development-related factors in monocots.

Within floral organs, the expression of *NtAGL6* in the single-petal cultivar showed a gradient of outer petals > ovary > stamens > pistils ≈ corona. This gradient partially overlaps with, but is not completely identical to, the typical expression pattern of A-class genes (highly expressed in sepals and petals, absent in carpels). Its expression in the ovary suggested that its functional boundary may be broader than that of typical A-class genes. In the double-petal cultivar, the expression levels of *NtAGL6* in petalized stamens and petalized pistils were significantly higher than those in the single-petal cultivar. However, this correlation alone cannot establish a causal relationship. Whether the petaloid phenotype is the cause or consequence of *NtAGL6* upregulation, or whether both are co-regulated by upstream factors, requires further distinction through loss- or gain-of-function experiments in the endogenous *Narcissus* system.

At the transcriptional regulation level, the *NtAGL6* promoter contained GA_3_-responsive elements (GARE-motif) and was significantly induced by exogenous GA_3_, which is consistent with the known role of the GA pathway in flowering regulation [37]. More notably, the promoter was significantly induced by high temperature. Since floral bud differentiation in *N. tazetta* var. *chinensis* requires a temperature above 25 °C [28], three independent lines of evidence from this study—the elevated expression of *NtAGL6* during flower bud differentiation, the presence of multiple HSE elements in its promoter, and the activation of GUS activity by high temperature—collectively point to a coherent working model: high temperature signals may promote floral transition by activating *NtAGL6* transcription. However, this inference remains indirect at present and awaits functional validation in the endogenous Narcissus system.

### 3.3. NtAGL6 Exhibits Flowering-Promoting Activity and Partial A-Class Functional Complementation in a Heterologous System

Ectopic expression of *NtAGL6* in wild-type *Arabidopsis* led to early flowering, accompanied by upregulation of *FT*, *SOC1*, *LFY*, and *AP1*, with *FT* showing the most significant increase. In the *ap1* mutant background, *NtAGL6* partially restored petals and sepals and induced sepal petalody in some flowers. These phenotypes suggested that *NtAGL6* can promote flowering and partially compensate for A-class floral organ identity determination when ectopically expressed in the heterologous *Arabidopsis* system.

This dual phenotype is not unique to *Narcissus*; ectopic expression of *HoAGL6* from hyacinth [32] and *PmAGL6* from mei flower [33] also produced both early flowering and floral homeotic transformation phenotypes. In *Arabidopsis*, endogenous *AGL6* primarily functions in flowering promotion [16,17], with A-class function being carried out by AP1 and AP2. In contrast, *AGL6* homologs from monocots such as *Narcissus* and hyacinth exhibit dual activities in heterologous systems. One possible explanation is that AGL6 proteins possess the molecular potential to interact with multiple MADS-box proteins. In the endogenous network of *Arabidopsis*, their interaction partners may be largely confined to the flowering time regulation pathway, whereas in plants such as *Narcissus*, they may have retained the ability to interact with proteins involved in floral organ identity determination. The observation that the expression level of the A-class gene *NtAP1* in sepals and petals of *N. tazetta* var. *chinensis* is much lower than that of *AP1* in *Arabidopsis* (our unpublished data) is consistent with this interpretation—when *AGL6* is still able to interact with floral organ identity proteins, the system’s demand for A-class gene expression may be relatively reduced.

However, a clear distinction must be made between “heterologous complementation capacity” and “endogenous function”. The restoration of petals and sepals in the *ap1* mutant by *NtAGL6* may result from heterologous interactions with *Arabidopsis* B-, C-, and E-class MADS-box proteins in the heterologous background, thereby compensating for the loss of AP1. This phenomenon indicates that *NtAGL6* possesses the molecular potential to interact with proteins related to floral organ identity determination, but it does not directly prove that it performs A-class function endogenously in *Narcissus*.

Regarding flowering promotion, the significant upregulation of *FT* by NtAGL6 is particularly noteworthy. *FT* is a central integrator of flowering time regulation. The nearly 29-fold upregulation of *FT* upon *NtAGL6* ectopic expression, far exceeding the upregulation of *SOC1*, *LFY*, and *AP1*, suggested that the early flowering phenotype may be mediated primarily through the *FT* pathway. This naturally raises the question: in what manner does *NtAGL6* regulate *FT*?

### 3.4. Regulation of NtFT1 by NtAGL6 May Occur Through an Indirect Pathway

The direct answer to the above question is as follows: dual-luciferase assays showed that *NtAGL6* could increase the LUC activity driven by the *NtFT1* promoter by 2.57-fold, but yeast one-hybrid assays detected no direct binding of *NtAGL6* to the *NtFT1* promoter. This combined result of “activation without binding” suggested that the regulation of *NtFT1* by *NtAGL6* may occur through an indirect pathway. The *NtFT1* gene used in this study was previously identified as the functional *FT* homolog in *N. tazetta* var. *chinensis* through sequence homology, phylogenetic analysis, and expression profiling [38].

This finding directly addresses the core question raised in the Introduction. In *Arabidopsis*, *AGL6* has been reported to promote flowering through positive regulation of *FT* expression [16], whereas *NtAGL6*, although similarly activating the expression of an *FT* homolog in a heterologous system, may employ a different molecular pathway. This suggests that the AGL6-FT regulatory module may have adopted different cis-regulatory logics between monocots and dicots. Interestingly, even in *Arabidopsis*, recent studies have shown that *AGL6* can also regulate *FT* expression indirectly through repression of *ELF3* [19], indicating that the regulatory relationship between *AGL6* and *FT* is more complex than simple direct binding. Our finding that *NtAGL6* activates *NtFT1* without direct promoter binding may thus represent an alternative mode within the *AGL6*-*FT* regulatory module.

Regarding the molecular basis of this indirect regulation, we propose three non-mutually exclusive hypotheses: (1) *NtAGL6* may need to form heterodimers with unknown MADS-box proteins to bind DNA effectively, and alone lacks sufficient affinity for the CArG-box elements in the NtFT1 promoter; (2) *NtAGL6* may indirectly upregulate *FT* by repressing an unknown negative regulator of *FT*, such as a member of the *TFL1* family or a specific miRNA. Although no typical FLC homolog was retrieved in our preliminary transcriptome analysis of *N. tazetta* var. *chinensis*, the possibility of functionally equivalent negative regulators of *FT* cannot be excluded; (3) *NtAGL6* may recruit histone-modifying enzymes or chromatin remodeling complexes to alter the epigenetic state of the *FT* chromatin region [39,40]. This mechanism does not require direct contact of *NtAGL6* with DNA but would not be detected in the yeast one-hybrid system. Among these three hypotheses, the first is particularly worthy of attention, as heterodimer formation among MADS-box proteins is a well-known phenomenon [13,41], and studies have shown that AGL6 proteins possess the structural basis for interacting with other MADS-box proteins.

Regardless of which mechanism is ultimately confirmed, the difference in *FT* regulatory mode between *NtAGL6* and *Arabidopsis AGL6* itself suggests that the connection mode of the AGL6-FT regulatory module is plastic during plant evolution. Although in vitro assays such as EMSA could provide further validation in the future, the current Y1H data, combined with the bioinformatic analysis of the promoter, strongly point to the dominance of an indirect regulatory pathway. It should also be noted that the dual-luciferase assay was performed in a heterologous system, which may not fully recapitulate the endogenous regulatory context.

### 3.5. Limitations and Future Directions

The main limitation of this study lies in the functional validation platform. Because a genetic transformation system for *N. tazetta* var. *chinensis* is not yet available, gene function validation relied entirely on heterologous expression in *Arabidopsis* and transient expression in tobacco. The fundamental limitation of this strategy is that the flowering-promoting and A-class complementary phenotypes observed for *NtAGL6* in *Arabidopsis* may partially result from heterologous interactions with *Arabidopsis* MADS-box proteins; similarly, the promoter activation effects observed in tobacco leaves cannot be fully equated with the endogenous regulatory environment in *Narcissus* floral meristems. Therefore, the functional inferences regarding *NtAGL6* in this study are preliminary and await verification in the endogenous *Narcissus* system. The 803 bp promoter fragment used in this study contains all core elements and key regulatory motifs relevant to our treatments and was experimentally confirmed to drive transcriptional activity. Nevertheless, we acknowledge that longer distal regions beyond this fragment may contain additional regulatory elements, although a longer fragment could not be obtained due to the current lack of a complete genome assembly for *N. tazetta* var. *chinensis*. Additionally, the specific molecular mediators involved in the indirect regulation of FT have not yet been identified, representing the most critical unresolved question at present.

Future research may be directed toward the following areas: (1) establishing a transient transformation system for *Narcissus* protoplasts or a VIGS (virus-induced gene silencing) system to validate the function of *NtAGL6* and its regulatory relationship with *FT* in the native *Narcissus* background; (2) using yeast two-hybrid library screening or co-immunoprecipitation combined with mass spectrometry to systematically identify protein partners that interact with *NtAGL6*, with particular focus on bridging proteins that may mediate its indirect association with the *FT* promoter; and (3) integrating the promoter analysis results to further dissect how temperature signals are transmitted through the NtAGL6-FT module to the flowering decision center, thereby elucidating at the molecular level the unique mechanism of high temperature-induced flowering in *N. tazetta* var. *chinensis*.

## 4. Materials and Methods

### 4.1. Plant Materials and Growth Conditions

**Figure 8:**
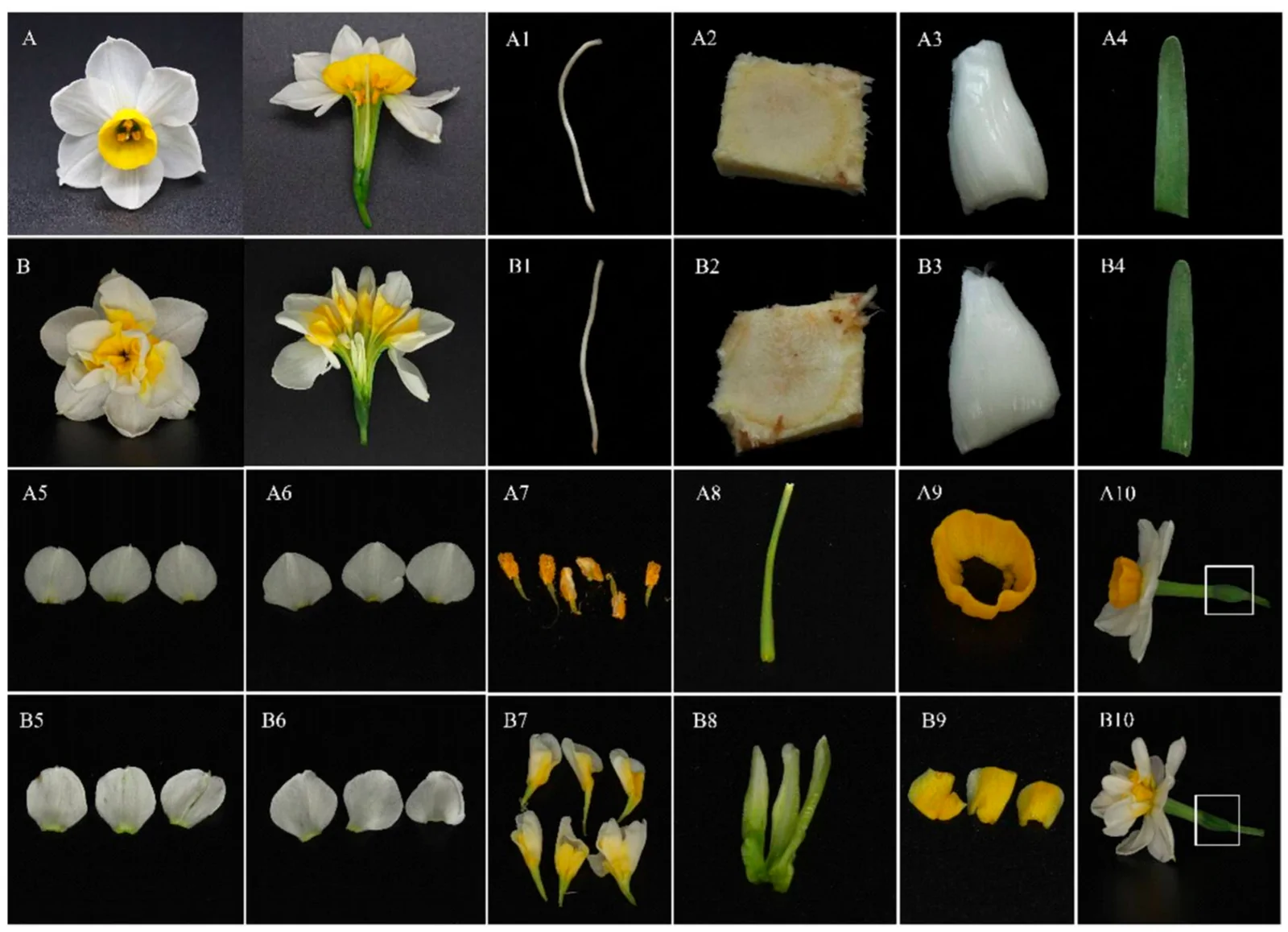
Different tissues of single and double narcissus. (**A**): Single flower; (**A1**–**A10**): root, basal plate, scale, leaf, tepal 1 (outer petal), tepal 2 (inner petal), stamen, pistil, corona, whole flower. (**B**): Double flower; (**B1**–**B10**): root, basal plate, scale, leaf, sepal (outer petal), petal, petalized stamen, petalized pistil, corona, whole flower. The boxed region indicates the ovary.

Three-year-old bulbs of *N. tazetta* var. *chinensis* Roem cultivars ‘Jinzhan Yintai’ (single-petal) and ‘Yulinglong’ (double-petal) were purchased from Zhangzhou, Fujian, China. The bulbs were cleaned of old roots, disinfected, and then grown hydroponically in water at 25 °C under natural light conditions. The water was changed regularly during hydroponic culture. After roots and leaves had grown to appropriate stages, ten tissue types were collected: root, basal plate, scale, leaf, sepal (outer tepal), petal (inner tepal), stamen, pistil, corona, and ovary. These samples were used for tissue-specific expression analysis. The anatomical structures of the floral organs are shown in Figure 8.

Samples for flower bud differentiation stages were collected from ‘Jinzhan Yintai’ bulbs stored at room temperature. The main buds were dissected periodically and classified into four stages based on morphological criteria: S1 (leaf bud stage, vegetative growth), S2 (inflorescence primordium formation stage), S3 (floral primordium formation stage, with individual flower bud primordia emerging within the spathe), and S4 (corolla formation stage, with floral organ primordia differentiating). Samples were collected at each stage. All collected samples were immediately frozen in liquid nitrogen and stored at −80 °C for subsequent RNA extraction.

Seeds of *Arabidopsis thaliana* wild-type Columbia (Col-0) and the mutant *ap1* (SALK_151561C, Col-0 background) were obtained from the *AraShare Arabidopsis* Mutant Stock Center. Seeds were stratified at 4 °C for 3 d and then sown in nutrient soil (peat:vermiculite = 3:1, *v*/*v*). Plants were grown in a controlled environmental growth chamber under long-day conditions (16 h light/8 h dark) at 22 °C, with a light intensity of 100 μmol·m^−2^·s^−1^ and relative humidity of 60–70%.

Seeds of *Nicotiana benthamiana* were sown in nutrient soil and grown under the same long-day conditions (22 °C, 16 h light/8 h dark, 100 μmol·m^−2^·s^−1^) for dual-luciferase transient expression assays and promoter GUS activity detection.

### 4.2. Total RNA Extraction and cDNA Synthesis

Approximately 100 mg of each tissue sample stored at −80 °C was ground into a fine powder in liquid nitrogen. Total RNA was extracted using the FastPure Plant Total RNA Kit (TaKaRa Bio Inc., Dalian, China) following the manufacturer’s instructions. The quality and concentration of total RNA were assessed by 1.0% agarose gel electrophoresis and a NanoDrop 2000 spectrophotometer (Thermo Fisher Scientific, Waltham, MA, USA), respectively. For reverse transcription, 1 μg of total RNA was used with the HiScript^®^ II 1st Strand cDNA Synthesis Kit (Vazyme Biotech Co., Ltd., Nanjing, China). The synthesized cDNA was stored at −20 °C for further use.

### 4.3. Cloning of the NtAGL6 Gene

Based on the transcriptome database of *N. tazetta* var. *chinensis* previously obtained by our research group (unpublished data), *AGL6* homologous sequences were screened through keyword searches and sequence alignment. Specific primers NtAGL6-F and NtAGL6-R (primer sequences listed in Appendix A Table A1) were designed according to the open reading frame (ORF) of the target sequence. PCR amplification was performed using the high-fidelity DNA polymerase PrimeSTAR^®^ HS DNA Polymerase (TaKaRa) with cDNA from petals of ‘Jinzhan Yintai’ as template. The purified products were ligated into the pMD™18-T Vector (TaKaRa) and transformed into *Escherichia coli DH5α* competent cells. After identification by colony PCR, positive clones were selected and sent to Sangon Biotech (Shanghai, China) for sequencing.

### 4.4. Sequence Alignment and Phylogenetic Analysis

The sequenced *NtAGL6* sequence was submitted to the NCBI database for BLAST homology searches (https://blast.ncbi.nlm.nih.gov/Blast.cgi, accessed on 15 May 2025). AGL6 homologous protein sequences from representative species, including *Arabidopsis*, rice, maize, wheat, orchid, magnolia, mei flower (*Prunus mume*), and hyacinth, as well as protein sequences of other MADS-box subfamily members such as AP1 and SEP, were downloaded. Multiple sequence alignment was performed using the Clustal W (version 2.1) program. A phylogenetic tree was constructed using MEGA 7.0 software with the Neighbor-Joining (NJ) method. The p-distance model was selected, gaps were handled by pairwise deletion, and bootstrap analysis was performed with 1000 replicates.

### 4.5. Quantitative Real-Time PCR (qPCR)

Specific primers qNtAGL6-F and qNtAGL6-R were designed based on the specific region of the *NtAGL6* cDNA. The *Narcissus Actin* gene (GenBank: JN204912) was used as the internal reference gene for *N. tazetta* var. *chinensis*, and *Arabidopsis ACT2* (GenBank: NM_001338358) was used for *Arabidopsis thaliana*. Pre-experiments confirmed that the *Actin* was stably expressed across all tested tissues. Primer sequences are listed in Appendix A Table A1. qPCR reactions were performed using Hieff^®^ qPCR SYBR^®^ Green Master Mix (No Rox) (Yeasen Biotechnology Co., Ltd., Shanghai, China) on a Bio-Rad CFX96™ Real-Time System (Bio-Rad Laboratories, Hercules, CA, USA). Each sample included three biological replicates. Relative expression levels were calculated using the 2^−ΔΔCt^ method, and data are presented as means ± standard deviation (SD). Statistical significance among multiple groups was determined by one-way ANOVA followed by Duncan’s multiple range test (*p* < 0.05). Different lowercase letters above the bars indicate significant differences.

### 4.6. Cloning and Analysis of the NtAGL6 Promoter

Genomic DNA was extracted from leaves of ‘Jinzhan Yintai’ using the DNAprep Pure Plant Kit (Tiangen Biotech Co., Ltd., Beijing, China). Based on the 5′-upstream sequence of the *NtAGL6* in the transcriptome database, nested PCR primers AGL6QDZ1, AGL6QDZ2, and AGL6QDZ3 (sequences listed in Appendix A Table A1) were designed. After three rounds of PCR using the Genome Walking Kits (TaKaRa). The cloned promoter sequence was analyzed for cis-acting regulatory elements using the online promoter analysis tools PlantCARE (http://bioinformatics.psb.ugent.be/webtools/plantcare/html/, accessed on 15 May 2025) and PLACE (http://www.dna.affrc.go.jp/PLACE/signalscan.html, accessed on 15 May 2025).

### 4.7. Construction of the GUS Expression Vector and Agrobacterium Transformation

The cloned *NtAGL6* promoter fragment was inserted into the plant expression vector pBI121 using the In-Fusion^®^ HD Cloning Kit (TaKaRa), replacing the original CaMV 35S promoter to generate the recombinant vector pBI121-NtAGL6::GUS. The recombinant plasmid was extracted and introduced into *Agrobacterium tumefaciens* strain GV3101 by the freeze–thaw method. Positive transformants were selected on YEP plates supplemented with 50 mg·L^−1^ kanamycin and 50 mg·L^−1^ rifampicin. Transformants were verified by colony PCR and then preserved for further use.

### 4.8. Transient GUS Expression Analysis of the Promoter

A single colony of *Agrobacterium* GV3101 harboring the recombinant plasmid pBI121-NtAGL6::GUS was inoculated into 5 mL of YEP liquid medium and cultured overnight at 28 °C with shaking at 200 r·min^−1^. The next day, the culture was diluted 1:50 into fresh YEP medium and grown until OD_600_ reached 0.6–0.8. The cells were collected by centrifugation at 6000 rpm for 10 min, and the supernatant was discarded. The pellet was resuspended in infiltration buffer (10 mmol·L^−1^ MgCl_2_, 10 mmol·L^−1^ MES, 150 μmol·L^−1^ acetosyringone, pH 5.6) and adjusted to OD_600_ = 0.8. The suspension was then incubated at room temperature for 2–3 h. *Agrobacterium* GV3101 without the plasmid was used as a negative control, while *Agrobacterium* carrying the empty pBI121 vector (OD_600_ adjusted to 0.8) served as a positive control.

Fully expanded leaves from the middle part of *Nicotiana benthamiana* plants were selected for infiltration. The bacterial suspension was injected into the abaxial side of the leaves using a 1 mL syringe (without needle). For each treatment, at least five plants were infiltrated, with 2–3 leaves per plant. After infiltration, the plants were placed in a light incubator at 22 °C under normal growth conditions.

The treatment scheme consisted of four groups: (1) Abiotic stress group: after 24 h of normal culture post-infiltration, plants were subjected to 35 °C for 48 h or 4 °C for 48 h. (2) Hormone treatment group: after 24 h of normal culture post-infiltration, plants were sprayed with 100 μmol·L^−1^ GA_3_, 100 μmol·L^−1^ ABA, or 100 μmol·L^−1^ MeJA, respectively. The sprays were applied every 12 h for a total of four sprays (treatment duration: 48 h). (3) Normal temperature control group: plants were cultured normally and sprayed with an equal volume of sterile water. (4) Positive control group: plants were transformed with the empty pBI121 vector (OD_600_ = 0.8) and cultured normally. All treatments were sampled at 72 h post-infiltration (i.e., 48 h after treatment initiation). Leaf disks approximately 1 cm in diameter around the infiltration site were collected for GUS staining.

GUS staining was performed according to the method described by Jefferson et al. [42]. Leaf disks were immersed in GUS staining solution (50 mmol·L^−1^ sodium phosphate buffer pH 7.0, 10 mmol·L^−1^ Na_2_EDTA, 0.5 mmol·L^−1^ K_3_[Fe(CN)_6_], 0.5 mmol·L^−1^ K_4_[Fe(CN)_6_], 0.1% Triton X-100, and 1 mmol·L^−1^ X-Gluc, freshly prepared) and incubated at 37 °C in the dark for 12–16 h. After staining, the disks were decolorized sequentially with 70% ethanol, 90% ethanol, and absolute ethanol until the green background was completely removed. The samples were observed and photographed under a stereomicroscope (Olympus SZX16). At least 15 leaf disks were observed for each treatment, and the experiment was independently repeated three times.

### 4.9. Subcellular Localization Vector Construction and Transient Expression in Onion Epidermal Cells

The complete ORF of *NtAGL6* (without the stop codon) was inserted into the pCAMBIA1302 vector (containing an mGFP tag) using the In-Fusion method to construct the 35S::NtAGL6-GFP fusion expression vector. The recombinant plasmid was verified by sequencing and then transformed into *Agrobacterium* GV3101.

Fresh onion (*Allium cepa* L.) bulbs were peeled of the outer 3–4 layers of scales and surface-sterilized by soaking in 75% ethanol for 10 min, followed by four washes with sterile water. Using a sterile scalpel, approximately 1 cm × 1 cm squares were marked on the inner epidermis, which was then carefully peeled off with forceps. The epidermal strips were placed on MS solid medium (containing 3% sucrose and 0.8% agar, pH 5.8) and pre-cultured at 25 °C in the dark for 20 h.

*Agrobacterium* suspensions containing 35S::NtAGL6-GFP or the empty pCAMBIA1302 vector (OD_600_ = 0.8) were prepared following the method described in Section 4.8. The pre-cultured onion epidermal strips were immersed in the bacterial suspension for 15 min, then removed and blotted dry with sterile filter paper. The strips were placed on MS solid medium and cultured at 25 °C in the dark for 2 d. After culture, the epidermal strips were washed three times with sterile water, mounted on glass slides with coverslips, and observed under a laser scanning confocal microscope (Leica TCS SP8, Wetzlar, Germany) for GFP fluorescence signals. The excitation wavelength was 488 nm, and emission was detected at 505–530 nm. Onion epidermal cells transformed with the empty pCAMBIA1302 vector served as controls to observe the intracellular distribution of GFP. The experiment was independently repeated three times, and no fewer than 20 positive cells were observed per replicate.

### 4.10. Arabidopsis Transformation and Screening of Transgenic Lines

The recombinant vector 35S::NtAGL6 was transformed into *Agrobacterium* GV3101 by the freeze–thaw method. *Arabidopsis* transformation was performed using the floral dip method described by Clough and Bent [43]. Wild-type Col-0 and *ap1* mutant *Arabidopsis* plants were grown until bolting and flowering. Open flowers and mature siliques were removed to promote the growth of lateral inflorescences. When the lateral inflorescences were at full bloom, the *Agrobacterium* suspension was prepared as described above to OD_600_ = 0.8, and Silwet L-77 was added to a final concentration of 0.02%. The inflorescences were dipped into the bacterial suspension for 30 s with gentle agitation, then removed, covered with a plastic bag to maintain high humidity, and kept in the dark for 24 h, followed by normal culture. The transformation was repeated once after 7 d. Plants transformed with the empty pCAMBIA1302 vector served as controls.

T_0_ seeds were harvested, sterilized with 70% ethanol for 1 min and 2.5% sodium hypochlorite for 10 min, then rinsed five times with sterile water. The seeds were sown on MS selection medium supplemented with 20 mg·L^−1^ hygromycin, stratified at 4 °C for 3 d, and then transferred to long-day conditions at 22 °C. After 7–10 days of selection, resistant seedlings (with normal root elongation and green cotyledons) were transplanted into nutrient soil. T_1_ plants were further screened on hygromycin-containing medium, and lines showing a segregation ratio of approximately 3:1 were considered to carry a single-copy insertion. Homozygous transgenic lines were obtained by T_3_ generation. At least three independent homozygous lines per transformation event were used for subsequent phenotypic analysis. Transgenic plants were verified by PCR using NtAGL6-specific primers and by qRT-PCR for expression level analysis before use in experiments.

### 4.11. Phenotypic Observation and Flowering Time Measurement of Transgenic Arabidopsis

T_3_ homozygous transgenic Arabidopsis seeds (35S::NtAGL6/Col-0, 35S::NtAGL6/ap1, and their respective empty vector controls) were stratified at 4 °C for 3 d and then sown in nutrient soil. Plants were grown under long-day conditions at 22 °C. The number of days from sowing to bolting (when the inflorescence stem elongated to approximately 1 cm) was recorded daily for each plant. The experiment was independently repeated three times. Flowering time data are presented as means ± SD, and statistical significance was analyzed using Student’s *t*-test (* *p* < 0.05, ** *p* < 0.01).

At bolting stage, inflorescences of *Arabidopsis* were observed and photographed under a stereomicroscope (Olympus SZX16) to document floral organ morphology. Particular attention was paid to the rescue of petal and sepal phenotypes in transgenic plants of the *ap1* mutant background, as well as any homeotic transformation phenotypes.

### 4.12. Expression Analysis of Flowering-Related Genes in Transgenic Arabidopsis

Rosette leaves and shoot apical meristem tissues were collected from 35S::NtAGL6/Col-0 transgenic *Arabidopsis* and empty vector control plants. Total RNA was extracted and cDNA was synthesized following the method described in Section 4.2. The *Arabidopsis Actin* gene was used as the internal reference. The expression levels of key flowering regulatory genes *FT*, *SOC1*, *LFY*, and *AP1* were examined. Primer sequences are listed in Appendix A Table A1, and the qPCR system and program were the same as described in Section 4.5. Three biological replicates were performed for each line, and the empty vector control was used as the calibrator sample to calculate the relative expression levels of each gene.

### 4.13. Dual-Luciferase Reporter Assay

The promoter fragment of the *NtFT1* gene was inserted into the multiple cloning site of the pGreenII 0800-LUC vector using the In-Fusion method to construct the reporter vector NtFT1Pro-LUC, which contains the firefly luciferase gene (LUC) and the internal control Renilla luciferase gene (REN). Meanwhile, the complete ORF of *NtAGL6* was inserted into the pGreenII 0800-62-SK vector to construct the effector vector 35S::NtAGL6. All recombinant plasmids were verified by sequencing and then transformed into *Agrobacterium* GV3101.

*Agrobacterium* strains harboring the reporter and effector vectors were inoculated separately into YEP liquid medium and cultured as described above to OD_600_ = 0.6–0.8. The cells were collected by centrifugation at 4000 rpm for 10 min, resuspended in infiltration buffer, and adjusted to OD_600_ = 0.8. The *Agrobacterium* cultures containing the reporter vector and the effector vector were mixed at a 1:1 volume ratio and then infiltrated into leaves of *Nicotiana benthamiana* plants. Co-infiltration of the reporter vector with *Agrobacterium* carrying the empty pGreenII 0800-62-SK vector served as the control.

After infiltration, the tobacco plants were grown under long-day conditions at 22 °C for 72 h. Leaf disks approximately 0.8 cm in diameter were collected from the edge of the infiltration zone (avoiding the central injection point) using a hole puncher and immediately frozen in liquid nitrogen. Samples were processed according to the protocol of the Dual Luciferase Reporter Gene Assay Kit (Promega, Madison, WI, USA). Firefly luciferase (LUC) and Renilla luciferase (REN) luminescence were measured sequentially using a microplate reader (SpectraMax iD3, Molecular Devices). The LUC/REN ratio was calculated to reflect promoter activity. Each treatment included four biological replicates (four leaves), and the experiment was independently repeated three times. Data are presented as means ± SD, and statistical significance was analyzed using Student’s *t*-test.

### 4.14. Yeast One-Hybrid Assay

The *NtFT1* promoter fragment (same as in Section 4.13 ) was inserted into the pHIS2 vector using the In-Fusion method to construct the bait vector pHIS2-proNtFT1. The complete ORF of NtAGL6 was inserted into the pGADT7 vector to construct the prey vector pGADT7-AGL6. The recombinant plasmids were verified by sequencing and then co-transformed into *Saccharomyces cerevisiae* strain Y187 using the PEG/LiAc method. Positive control (pGADT7-53 + p53-pHIS2) and negative control (pGADT7 + pHIS2-proNtFT1) were also included.

The transformed yeast cells were plated on SD/-Leu-Trp deficient solid medium and incubated at 30 °C for 3–5 d until colonies appeared. To determine the minimal inhibitory concentration of 3-amino-1,2,4-triazole (3-AT) required to suppress background autoactivation of the His3 reporter gene, yeast cells transformed with the empty pHIS2-proNtFT1 vector were first plated on SD/-Leu-Trp-His deficient medium containing 0, 5, 10, 15, 20, 25, 30, 35, 40, 45, or 50 mmol·L^−1^ 3-AT. After incubation at 30 °C for 5 d, the lowest 3-AT concentration that completely inhibited yeast growth (30 mmol·L^−1^) was selected for subsequent screening assays. Single colonies of the co-transformed experimental and control groups were picked, resuspended in sterile water, adjusted to OD_600_ = 0.1, and 5 μL of each suspension was spotted onto SD/-Leu-Trp-His deficient solid medium containing 30 mmol·L^−1^ 3-AT. The plates were incubated at 30 °C for 3–5 d, and yeast growth was monitored.

## 5. Conclusions

In this study, the *AGL6* homologous gene *NtAGL6* was cloned and identified from *N. tazetta* var. *chinensis*. This gene was specifically expressed in floral organs and was upregulated during flower bud differentiation. Its promoter region contained environmental signal-responsive elements, including heat shock and gibberellin elements. Heterologous functional analysis suggested that *NtAGL6* can promote flowering and partially compensate for A-class floral organ identity determination when ectopically expressed in *Arabidopsis*. More importantly, although *NtAGL6* could activate the transcription of its downstream gene *NtFT1*, it did not directly bind to the *NtFT1* promoter, suggesting that its regulation of *FT* may be achieved through an indirect pathway. Further validation in the endogenous *Narcissus system* and identification of the specific mediators involved remain important directions for future research.

## Data Availability

The data presented in this study are available on request from the corresponding author due to authorization rules of the corresponding author’s institution.

## Abbreviations

The following abbreviations are used in this manuscript:

3-AT	3-amino-1,2,4-triazole
ABA	Abscisic acid
DAPI	4′,6-diamidino-2-phenylindole
EMSA	Electrophoretic mobility shift assay
FT	FLOWERING LOCUS T
GA_3_	Gibberellin A_3_
GFP	Green fluorescent protein
GUS	β-glucuronidase
LUC	Firefly luciferase
LTRE	Low-temperature responsive element
MeJA	Methyl jasmonate
MS	Murashige and Skoog medium
ORF	Open reading frame
qRT-PCR	Quantitative real-time PCR
REN	Renilla luciferase
VIGS	Virus-induced gene silencing
YEP	Yeast extract peptone medium

## Appendix A

**Table A1:** Primers used in this study.

Primer Name	Sequence
**For PCR**	
NtAGL6-F	5′-ATGGGGAGAGGGAGAGTTGAATTG-3′
NtAGL6-R	5′-CTAAAGGACCCAACCCAGCATG-3′
AGL6QDZ1	5′-CTCAATGGGCTAAGGTCTTCTCC-3′
AGL6QDZ2	5′-GCCAGCACTCCCAAACTCATAGAGCTTCCC-3′
AGL6QDZ3	5′-CAATCCTCTTCAATTCAACTCTCCCTCTC-3′
pro NtAGL6-F	5′-CGGTCGCTCGCGTCGTGGAGTC-3′
pro NtAGL6-R	5′-CTTCCTCCTTTCCTTTTCTCT-3′
proNtFT1-F	5′-GACTCCAGAGCGGCCGCGTG-3′
proNtFT1-R	5′-CCAGCACATCACCAATTATCCTACC-3′
**For qPCR in** *** Narcissus tazetta*** ** var.** *** chinensis*** ** Roem**	
qNtAGL6-F	5′-GCGGTTGTAGGAAATGCATACTC-3′
qNtAGL6-R	5′-TAAGATCCAACAGAGTTCACCATGG-3′
qNtActin-F	5′-GTTGACCCACCACTAAGAACAATG-3′
qNtActin-R	5′-TGCCCAGAAGTGCTATTCCAG-3′
**For promoter activity analysis**	
pBI121-proAGL6-F	5′-GACCATGATTACGCCAAGCTTCGGTCGCTCGCGTCGTGGAGTC-3′
pBI121-proAGL6-R	5′-GAGAACACGGGGGACTCTAGACTTCCTCCTTTCCTTTTCTCT-3′
**For stable** *** Arabidopsis thaliana*** ** transformation**	
pCAMBIA1302-NtAGL6-F	5′-ACTCTTGACCATGGTAGATCTATGGGGAGAGGGAGAGTTG-3′
pCAMBIA1302-NtAGL6-R	5′-TCTCCTTTACTAGTCAGATCTAAGGACCCAACCCAGC-3′
**For qPCR in transgenic** *** Arabidopsis thaliana***	
AtActin-F	5′-GCAGCATGAAGATCAAGGTGG-3′
AtActin-R	5′-GGTAACAAACTCACCACCACG-3′
AtSOC1-F	5′-CTCTCAGTGCTTTGTGATGCT-3′
AtSOC1-R	5′-CGATTGAGCATGTTCCTATGCC-3′
AtFT-F	5′-GCGCCAGAACTTCAACACTC-3′
AtFT-R	5′-TAGGCATCATCACCGTTCGT-3′
AtLFY-F	5′-GTTAGGTTTTACGGCGAGCA-3′
AtLFY-R	5′-GCAATCGTCTCCGTTCAGC-3′
AtAP1-F	5′-ACCAAATCCAGCATCCTTACA-3′
AtAP1-R	5′-TCAAGAGTCAGTTCGAGATCATTC-3′
**For Dual luciferase assay**	
NtAGL6-62SK-F	5′-CGCGGTGGCGGCCGCTCTAGAATGGGGAGAGGGAGAGTTGAA-3′
NtAGL6-62SK-R	5′--TCAGCGTACCGAATTGGTACCCTAAAGGACCCAACCCAGCA-3′
pGreenII0800-proNtFT1-F	5′-GTCGACGGTATCGATAAGCTTGACTCCAGAGCGGCCGCGTG-3′
pGreenII0800-proNtFT1-R	5′-GGCGGCCGCTCTAGAACTAGTCCAGCACATCACCAATTAT CCTACC-3′
**For Yeast one-hybrid assay**	
pHIS2-proNtFT1	5′-ACTCACTATAGGGCG GACTCCAGAGCGGCCGCGTG-3′
pHIS2-proNtFT1	5′-CGATTCATCTGCAGCCCAGCACATCACCAATTATCC TACC-3′
pGADT7-AGL6	5′-CCATGGAGGCCAGTGAATTCATGGGGAGAGGGA GAGTTGAATTG-3′
pGADT7-AGL6	5′-AGCTCGAGCTCGATGGATCCCTAAAGGACCCAAC CCAGCATG-3′
